# Neutrophil to Lymphocyte Ratio and Cardiovascular Disease Incidence in HIV-Infected Patients: A Population-Based Cohort Study

**DOI:** 10.1371/journal.pone.0154900

**Published:** 2016-05-05

**Authors:** Eugenia Quiros-Roldan, Elena Raffetti, Francesco Donato, Michele Magoni, Chiara Pezzoli, Alice Ferraresi, Nigritella Brianese, Filippo Castelnuovo, Emanuele Focà, Francesco Castelli

**Affiliations:** 1 University Department of Infectious and Tropical Diseases, University of Brescia and Brescia Spedali Civili General Hospital, Brescia, Italy; 2 Unit of Hygiene, Epidemiology and Public Health, University of Brescia, Brescia, Italy; 3 Local Health Agency of the Brescia Province, Brescia, Italy; 4 Hospital Division of Infectious and Tropical Diseases, Brescia Spedali Civili General Hospital, Brescia, Italy; Scuola Superiore Sant'Anna, ITALY

## Abstract

Neutrophil to lymphocyte ratio (NLR) has been shown to predict occurrence of cardiovascular events in the general population. The aim of our study was to evaluate the role of NLR to predict major cardiovascular disease (CVD) events in HIV-infected subjects. We performed a retrospective cohort study of HIV-infected patients residing in the Local Health Authority (LHA) of Brescia, northern Italy, from 2000 to 2012. The incidence of CVD events in HIV-positive patients was compared with that expected in the general population living in the same area, computing standardized incidence ratios (SIRs). To evaluate the predictive role of NLR, univariate and multivariate Cox regression models were applied, computing hazard ratios (HRs). A total of 3766 HIV-infected patients (mean age 38.1 years, 71.3% males) were included (person-years 28768.6). A total of 134 CVD events occurred in 119 HIV-infected patients. A 2-fold increased risk (SIR 2.02) of CVD was found in HIV-infected patients compared to the general population. NLR levels measured at baseline and during follow-up were independently associated with CVD incidence, when also adjusting for both traditional CVD risk factors and HIV-related factors (HR 3.05 for NLR≥ 1.2). The area under the receiver operating characteristics (ROC) curve showed a modest, not statistically significant, increase, from 0.81 to 0.83, with addition of NLR to Framingham risk score model covariates. In conclusion an elevated NLR is a predictor of risk CVD in HIV-infected patients, independently from the traditional CVD risk factors.

## Introduction

A higher risk of cardiovascular disease (CVD) has been found in HIV infected than uninfected persons in a large USA cohort study [1]. After adjusting for Framingham risk factors, comorbidities, and substance use, HIV-positive subjects had a 1.5–2 fold increased risk of incident myocardial infarction compared with uninfected subjects, and the excess risk remained among those achieving an HIV-1 RNA level less than 500 copies/mL [2].

Hence, modifiable risk factors for CVD should be investigated in HIV infected patients, and lifestyle and pharmacological interventions should be undertaken as necessary. In routine HIV clinical care, it is recommended to calculate the CVD risk using consolidated prediction models [3]. However, the traditional cardiovascular risk assessment algorithms such as the Framingham Risk Score (FRS) were developed for the general population, and they have been shown to underestimate the CVD risk in HIV-infected subjects [4,5]. In fact, a specific algorithm, using the same variables of the FRS but new estimates of coefficients, was developed in a large multicenter cohort study (DAD cohort) on HIV-infected subjects [6,7].

However, these algorithms may not accurately predict CVD risk in HIV-infected patients because additional HIV-related risk factors, such as chronic inflammation and immunoactivation, are present even in patients with long-term effective combined antiretroviral therapy (cART) [8].

Recently, neutrophil to lymphocyte ratio (NLR) has been proposed as a simple and reliable systemic inflammatory marker, which has been shown to be associated with worse prognosis in patients with various CVDs [9, 10]. Moreover, NLR has been recently found to predict CVD mortality and to improve the discrimination, calibration and reclassification capability of the FRS model in a USA asymptomatic general population cohort [11]. NLR was also found useful to predict the mortality for non-AIDS defining cancer and for non-Hodgkin lymphoma in HIV subjects [12,13].

The aim of this study was to evaluate the role of NLR to predict CVD events in HIV infected subjects, when also taking account of the traditional risk factors for CVD.

## Materials and Methods

### Study population and data

We conducted a retrospective cohort study of HIV-infected patients, either naive or experienced to the cART, residing in the Local Health Authority (LHA) of Brescia province, northern Italy, receiving primary care for HIV infection at the Institute of Clinical Infectious Diseases of the Brescia “Spedali Civili” General Hospital. The period of enrolment and of follow-up was from January 2000 to December 2012. Inclusion criteria were: age of 18 years and over, and no CVD diagnosis before enrolment in the cohort. Socio-demographic characteristics, laboratory tests, treatments for HIV infection and clinical complications including CVD were recorded at enrolment and every 3–4 months, and stored in an ad hoc electronic database used for routine clinical management and research studies. Gender, age at enrolment, country of origin, HIV exposure group, period of enrolment in the cohort, Hepatitis C Virus (HCV) or Hepatitis B Virus (HBV) co-infection, diabetes mellitus and tobacco smoking were retrieved from the database. Furthermore, the following parameters measured at enrolment and every year of the follow-up, were collected: HIV-RNA, CD4 and CD8 cell counts, serum levels of total cholesterol, HDL and creatinine, neutrophil and platelet counts, systolic and diastolic blood pressure and antihypertensive therapy.

### Exposure and outcome variables

NLR was calculated as the ratio of neutrophil cell count to lymphocyte cell count. NLR showed an approximately log-normal distribution, and it was dichotomized using the value that maximized Youden's index for predicting CVD occurrence (maximum values of sensitivity plus specificity minus one) as cut-off value.

The main outcome of this study was incidence of CVD in the study period, including coronary death, myocardial infarction, coronary insufficiency, angina, ischemic stroke, hemorrhagic stroke, transient ischemic attack, peripheral artery disease and heart failure.

The CVD cases were retrieved from the following sources:

- the clinical charts and electronic database of the Institute of Clinical Infectious Diseases;
- the population-based CVD Registry of the Brescia LHA, through a record linkage based on each individual’s national registration code.

We identified 2 families of diseases using a set of ICD9-CM codes: Ischemic heart diseases (I20-I25 and I50) and intracerebral hemorrhage/cerebral infarction (I61-I66). An individual was considered to have and CVD if s/he had been hospitalized with those codes or deceased for a cause of death with one of those codes.

Information on vital status and date of death were obtained from a record-linkage with the LHA Mortality Registry. Presence of hypertension and diabetes mellitus were defined on the basis of data recorded in medical charts.

The study was conducted in accordance with the guidelines of the Declaration of Helsinki and the principles of Good Clinical Practice. The study protocol was approved by the Brescia Province Ethics Committee. Written informed consent was obtained by all patients enrolled.

### CUORE and Framingham Risk Score

The risk functions of the CUORE score (Cardiovascular risk score for the Italian general population), produced by the Italian National Institute of Health e Minister of Health, and that of the FRS were used for calculating the 10-year risk of CVD occurrence [14]. CVDs comprised coronary death, myocardial infarction, coronary insufficiency, angina, ischemic stroke, hemorrhagic stroke, transient ischemic attack, peripheral artery disease, heart failure.

The predictors considered in the CUORE score and FRS were: gender, age, diabetes, tobacco smoking, systolic blood pressure (SBP), presence of hypertension and/or antihypertensive treatment, total cholesterol and HDL cholesterol.

### Statistical analysis

The observation period ended either on 31 December 2012, or last follow-up visit, or death, or CVD occurrence, whichever occurred first during the study period. Median, mean, range and SD were used as descriptive statistics. In order to compare the incidence of CVD in HIV-infected patients to that observed in the general population living in the area, the observed-to-expected ratios of CVD (standardized incidence ratios, SIRs) and their corresponding 95% confidence intervals (95% CIs) were computed using the Byar’s approximation for the CIs [15]. The number of expected CVD cases was computed using the general population gender- and age-specific incidence rates provided by the Brescia LHA. 10-years cumulative incidences of first CVD event in HIV subjects according to categories of NLR were estimated using cumulative incidence function and compared using the Pepe–Mori test.

The association of NLR with occurrence of CVD first event was evaluated by univariate and multivariate analyses using both time independent and time dependent Cox proportional hazard models. To this end, five models were fitted: **Model A** included NLR; **Model B** included NLR, age, sex, hypertension, diabetes, tobacco smoking, SBP, total cholesterol and HDL; **Model C** included age, sex, diabetes and tobacco smoking as fixed covariates, and NLR, hypertension, SBP, total cholesterol and HDL as time dependent covariates; **Model D** included all variables in Model C plus intravenous drug use as fixed covariate and CD4 cell count and antiretroviral therapy as time dependent covariates; **Model E** included all variables in Model D plus glomerular filtration rate (GFR) estimated using the modification of diet in renal disease (MDRD) formula [16], as time dependent covariate. In Cox regression models including time dependent covariates, the study period was divided into intervals of 1-year duration, The results of these analyses were expressed as hazards ratios (HRs), their 95% CIs, and p-values according to Wald test.

To evaluate the shape of the association of NLR with CVD incidence, we also fitted multivariate time dependent Cox models with a cubic-spline for NLR. We used the Akaike’s information criterion (AIC) to choose the number of spline knots.

Two sensitivity analyses were also performed: (i) including NLR as a continuous variable in the models, and (ii) including the prescription of antiretroviral drugs associated with an increase of cardiovascular risk (lopinavir indinavir and abacavir) as a dichotomous covariate (yes/no) in the full model.

Moreover, receiver operating characteristics (ROC) curves were modeled in order to compare the capability of the CUORE score, of the risk score including CUORE/FRS covariates and of a risk score including CUORE/FRS covariates plus NLR to predict CVD occurrence. The areas under the curves (AUCs) and their 95% CIs are reported. The scores derived from the model including the CUORE/FRS covariates and from that including CUORE/FRS covariates plus NLR were computed using linear prediction equations from Cox proportional hazard models.

The proportional hazards assumption was investigated for each single covariate and globally by analyzing Schoenfeld residuals. We first produced the graphical plots and then carried out formal statistical tests of their independence over the rank transformation of time, but no departures from this assumption were found.

All statistical tests were two-sided, assumed a level of significance of 0.05 and were performed using Stata 12 software (Stata Statistics/Data Analysis 12.0—Stata Corporation, College Station, TX, USA).

## Results

### Patient characteristics

A total of 3766 HIV infected patients were enrolled, contributing 28768.6 person-years (median follow-up of 8.3 years). The cumulative probability of loss to follow-up at 3 years after enrolment was 5.2% (95% CI 4.5%-6.0%). The patients’ characteristics at enrolment are shown in Table 1. Mean age of the population was 38.1 years; 71.3% were males and 39.9% had intravenous drug use as the major risk factor for HIV acquisition. Among modifiable CVD risk factors, diabetes was found in 7.4%, hypercholesterolemia (total cholesterolemia >200 mg/dl) in 34.7%, hypertension in 7.9% and tobacco smoking in 65.6% of subjects.

**Table 1:** Demographical and clinical features at enrolment.

Variables	n(%)* (n = 3766)
**Male**	2685 (71.3)
**Age, in years, mean (SD)**	38.1 (8.9)
<40	1456 (38.7)
40–49	1635 (43.4)
≥50	675 (17.9)
**Intravenous Drug Use**	1434 (39.9)
**Immigrant**	484 (12.9)
**HBV/HCV co-infection**	1677 (47.2)
**cART**	2263 (60.1)
**CD4 cell count, cell/mm3, mean (SD)**	441.9 (267.1)
<200	610 (17.9)
200–349	780 (22.9)
350–499	824 (24.2)
≥500	1189 (34.9)
**CD4/CD8 ratio, mean (SD)**	0.49 (0.34)
<0.3	1158 (34.1)
0.3–0.45	710 (20.9)
≥0.45	1528 845.0)
**Positive HIV-RNA, > 37 copies/mL**	1919 (54.9)
**Tobacco smoking**	2040 (65.6)
**Diabetes**	279 (7.4)
**Triglycerides (mg/dl), mean (SD)**	162.0 (131.6)
≥ 150	1313 (39.2)
**Total Cholesterol (mg/dl), mean (SD)**	184.1 (56.3)
≥ 200	1160 (34.7)
**HDL Cholesterol (mg/dl)**	
<50 F <40 M	1018 (43.5)
**SBP (mm Hg), mean (SD)**	123.6 (15.4)
**Antihypertensive therapy**	70 (1.9)
**Hypertension**	299 (7.9)
**GFR (ml/min), mean (SD)**	108.4 (16.3)
**NLR, mean (SD)**	1.8 (1.2)
<1.2	1113 (32.2)
≥ 1.2	2341 (67.8)

*Colum percentage. Abbreviations: SD, standard deviation; cART, antiretroviral therapy; NLR, neutrophil to lymphocyte ratio; SBP, systolic blood pressure; HDL, high density lipoprotein; GFR, Glomerular filtration rate estimated using the Modification of diet in renal disease (MDRD) formula.

A total of 119 patients had a first CVD from 2000 to 2012: the Institute of Clinical Infectious Diseases electronic database identified 31 (26.0%) events, the LHA Registry 42 (35.3%) and both databases 46 (38.7%). The number of observed CVD incident cases was higher than the expected according to the Italian general population in both males (103 cases; SIR 1.96, 95%CI 1.62–2.34) and females (16 cases; SIR 2.42, 1.43–3.60). In the whole cohort, the observed-to-expected ratio of CVD events was 2.02 (1.69–2.37).

### Association between NLR blood levels and CVD incidence

At baseline, 3454 subjects had NLR measure, and the NLR distribution is shown in S1 Fig. The 25° percentile, median and 75° percentile were 1, 1.4 and 2.1, respectively. According to Youden's index, we used 1.2 as NLR cut-off. A total of 2341 (67.8%) subjects had NLR ≥1.2 at baseline.

**Fig 1:**
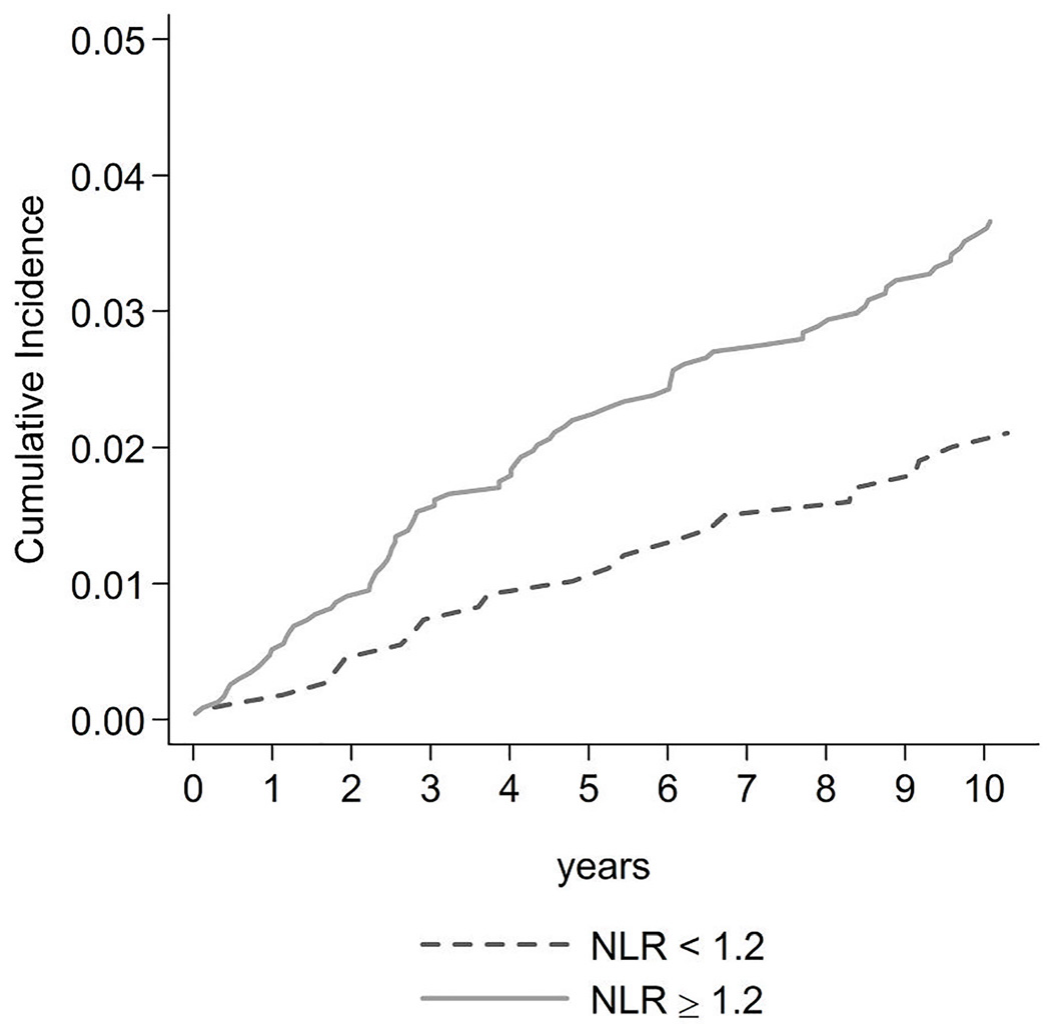
Cumulative incidence CVD curves in HIV-infected subjects with NLR<1.2 and NLR ≥1.2. Abbreviation: NLR, neutrophil to lymphocyte ratio.

Higher NLR was associated with greater cumulative incidence of first CVD event: a CVD event occurred in 22 of 1113 (2.0%) subjects with NLR<1.2 compared to 90 of 2341 (3.8%) subjects with NLR≥1.2 in the follow-up (chi square, p = 0.004). Fig 1 shows cumulative incidence curves for CVD according to NLR categories, with 10-years risks of CVD of 2.1% (standard error = 0.4%) and 3.6% (0.4%) for NLR < 1.2 and NLR ≥ 1.2, respectively (Pepe-Mori test, p = 0.003).

Univariate and multivariate Cox regression models showed that NLR was predictor of CVD, with HRs varying from 1.86 to 3.05 (Table 2), the relationship between NLR and CVD was statistical significant in four models and next to threshold in one model. These results did not change substantially when including the prescription of lopinavir, indinavir or abacavir as a covariate in the models.

**Table 2:** Hazard ratio for NLR ≥ 1.2 compared to NLR < 1.2 in predicting cardiovascular event incidence in five models.

Model	n	HR	95% CI	p value
**A**	3454	1.86	1.17–2.97	0.009
**B**	1980	2.16	1.05–4.44	0.036
**C**	2499	3.05	1.20–7.72	0.019
**D**	2436	2.94	1.16–7.48	0.023
**E**	1742	2.45	0.95–6.31	0.062

Model A included NLR.

Model B included NLR, age, sex, hypertension, diabetes, tobacco smoking, SBP, total cholesterol and HDL cholesterol.

Model C included age, sex, diabetes and tobacco smoking as fixed covariates, and NLR, hypertension, SBP, total cholesterol and HDL cholesterol as time dependent covariates.

Model D included Model C plus intravenous drug use as fixed covariate and CD4 cell count and antiretroviral therapy as time dependent covariates.

Model E included Model D plus GFR.

Abbreviations: HR, hazard ratio; 95% CI, 95% confidence interval; NLR, neutrophil to lymphocyte ratio; SBP, systolic blood pressure, HDL, high density lipoprotein; GFR, Glomerular filtration rate estimated using the Modification of diet in renal disease (MDRD) formula.

Incorporating NLR as a continuous variable in the five univariate and multivariate Cox regression models confirmed NLR as an independent predictor of CVD (S1 Table).

**Fig 2:**
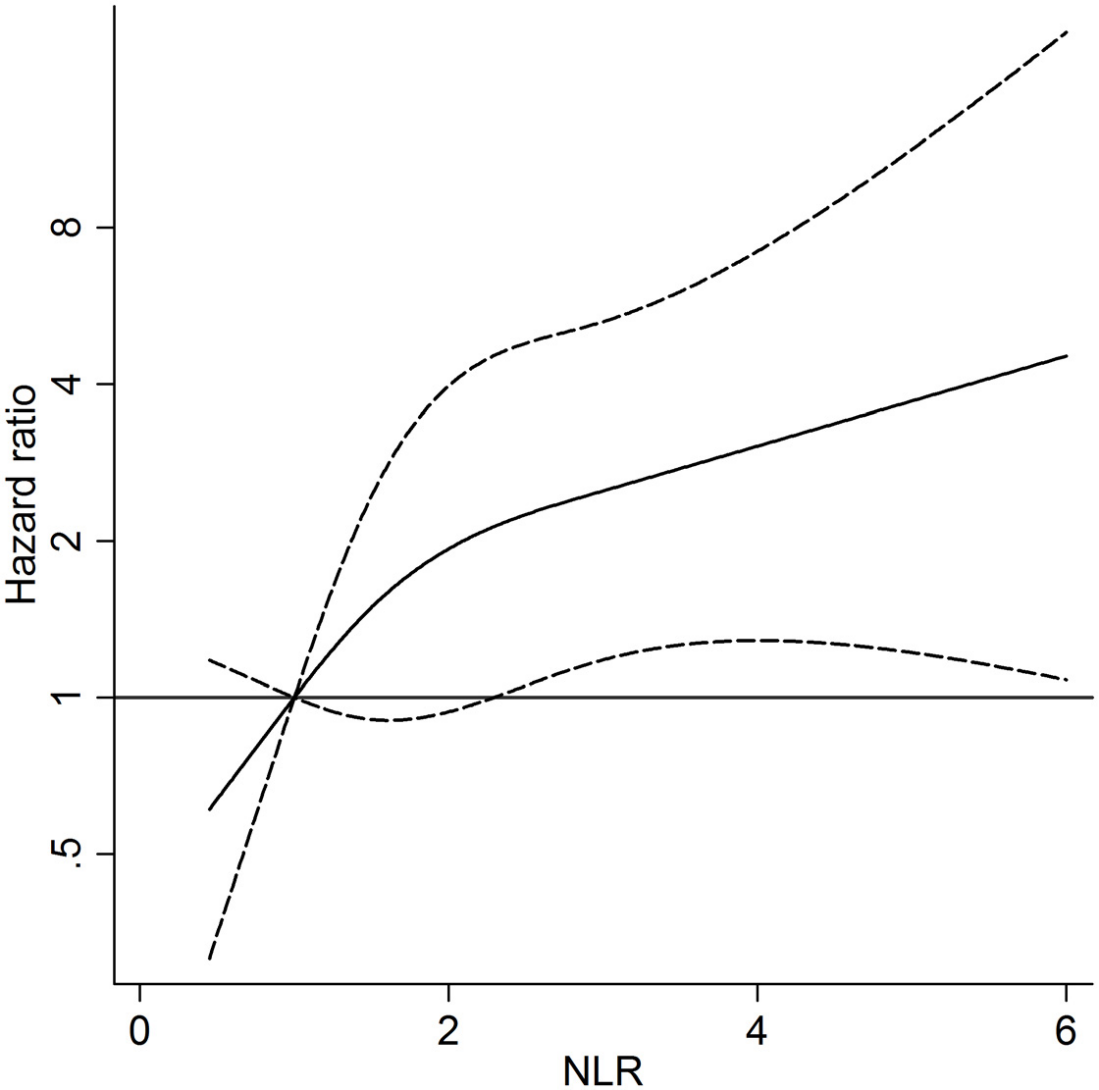
Risk of CVD event (Hazard Ratio) according to NLR distribution. NLR was modeled by cubic spline (solid line) with three knots in Cox regression model adjusted for age, sex, intravenous drug use, diabetes and tobacco smoking as fixed covariates, and CD4 cell count, antiretroviral therapy, presence of hypertension, SBP, total cholesterol and HDL as time dependent covariates. The reference value is 1.2. The 95% confidence intervals are shown as dashed lines. *Vertical axes* have a *logarithmic scale*. Abbreviations: NLR, neutrophil to lymphocyte ratio; SBP, systolic blood pressure; HDL, high density lipoprotein.

NLR was also evaluated in a multivariate time dependent Cox regression model with restricted cubic-splines for NLR, showing that the risk of CVD increased linearly with increasing NLR (Fig 2).

The AUCs for the CUORE score, and for the models including CUORE/FRS covariates and NLR plus CUORE/FRS covariates were 0.71, 0.81 and 0.83, respectively (Table 3).

**Table 3:** Areas under the ROC curve according to different risk scores of CVD incidence.

Score	Area under the ROC curve	95% CI
**CUORE risk score**	0.71	0.58–0.84
**CUORE/FRS covariates**	0.81	0.73–0.89
**CUORE/FRS covariates plus NLR**	0.83	0.75–0.91

Abbreviations: ROC, receiver operating characteristic; 95% CI, 95% confidence interval; FRS, Framingham risk score; NLR, neutrophil to lymphocyte ratio.

## Discussion

In the present study, based on a cohort of HIV-infected patients, the risk of CVD was around 2-fold higher in HIV-infected patients than in the general population living in the area (SIR = 2.02). The main and new finding of our study is that, in these subjects, NLR levels measured at baseline and during follow-up were associated with CVD incidence, independently from the classic CVD risk factors. This association remained significant when also adjusting for the HIV-related factors associated with cardiovascular events (CD4 lymphocytes number, HIV exposure category or antiretroviral drugs), with an adjusted HR up to 3.05 for NLR ≥ 1.2 in the model including traditional CVD risk factors. These findings are in agreement with other studies carried out in the general population. Particularly, the USA National Health and Nutrition Examination Survey-III (NHANES-III), showed that NLR was an independent predictor of CHD mortality in a general population cohort of subjects aged 30–79 years free from CHD at baseline, with an adjusted HR of 2.68 for NLR > 4.5 compared to NLR < 1.5 [11].

The systemic inflammation has been found to be associated with CVD risk in various studies, as it plays an important role in atherogenesis and atherothrombotic events [17]. Indeed, various inflammatory markers, particularly C-reactive protein (CRP), fibrinogen, albumin and leukocyte count have been shown to be predictors of CVD event occurrence [18–20].

Most HIV patients suffer from chronic inflammation, because of their disease status and cART [21]. Therefore, various biomarkers of inflammation, immune activation, and endothelial dysfunction, mainly IL-6, TNF and C-reactive proteine (CRP), have been found related with CVD incidence also in HIV patients [22,23].

The NLR is a ratio between peripheral neutrophil and lymphocyte counts. It is an emerging marker of systemic inflammation which has been demonstrated to predict mortality for several clinical conditions, including CVD [9–11]. Various mechanisms have been hypothesized for the association between NLR and CVD risk [24]. On the one hand, neutrophils, after emigration into vessel walls, produce proinflammatory and atherogenic effects and their recruitment is associated with plaque rupture [25,26]. On the other hand, low lymphocyte counts have been associated with worse outcomes in patients with CHD and unstable angina by itself, though mechanism is still unknown [27,28].

We confirmed that traditional CVD risk factors, such as age, diabetes, hypertension and tobacco smoking, were associated with CVD risk in HIV positive subjects, with HRs similar to those found in healthy people [29]. We found no association of dyslipidemia with CVD in our patients possibly because most of those with high levels of total serum cholesterol were under treatment with statins. Also HIV-related risk factors, including substance abuse, and cART, might contribute to increased risk of CVD in HIV-infected patients as shown in one study [6], and for this reason we included also these variables in some models. However, the inclusion of these HIV-related variables did not modify the results of our study.

In our study, when NLR was added to the model including FRS and CUORE variables, the performance of the new model improved only marginally, with the AUC increasing from 0.81 to 0.83, the difference being not statistically significant. It should be noted, however, that it is difficult to raise the AUC when the model including traditional risk factors already discriminates well in this cohort. Accordingly, there was no significant improvement in AUC with addition of NLR to Framingham risk score model in prediction of CHD mortality in the NHANES-III study [11]. These results are in line with those of other studies carried out to evaluate the clinical utility of adding biomarkers of inflammation to traditional CVD risk score models. For instance, a recent cohort study showed that addition of CRP to basic risk factor assessment was not clinically useful for identifying patients at risk of a first CHD event [30].

However, some evidence indicates that adding inflammation biomarkers such as CRP to risk prediction models may be useful among initially intermediate-risk persons [31]. Indeed, current recommendations of scientific societies advocate routine assessment of high-sensitivity CRP in healthy people with intermediate cardiovascular risk, as an optional screening test for deciding initiation of pharmacological therapy after quantitative risk assessment [32]. Accordingly, NLR evaluation in HIV patients might be of clinical utility for better evaluation of their CVD risk, because most of them are relatively young and at low-intermediate CVD risk.

The strengths of this study include the large population size, the enrolment of an unselected group of HIV-infected patients, and accurate retrieval of data on CVD events, due to the use of two different sources, the clinic database and the population-based LHA Registry. This study has also some limitations. First, we had no data on family history of CVD, which therefore was not included in the CVD risk score models. Second, the number of CVD events observed during a medium-term observation period was relatively low, due to the young age of our cohort at enrolment (mean age 38 years). This prevented us from performing analyses of the NLR contribution to traditional CVR risk score prediction models according to the levels of CVD risk (low, intermediate and high risk).

## Conclusions

In conclusion, we have shown that NLR is a predictor of CVD risk in HIV-infected patients, independently from the classic risk factors the disease. Due to the lack of other studies on this issue at present, these findings should be considered with caution and need to be confirmed. Furthermore, at present there is no evidence that changes in the level of markers of inflammation, such as CRP, NLR and others, be useful in primary prevention of CVD.

## Supporting Information

S1 Fig NLR distribution at enrollment. Abbreviation: NLR, neutrophil to lymphocyte ratio.
(TIF)

S1 Table Hazard ratio of CVD event for NLR as a continuous variable using various Cox proportion hazard models. Model A included NLR. Model B included NLR, age, sex, hypertension, diabetes, tobacco smoking, SBP, total cholesterol and HDL. Model C included age, sex, diabetes and tobacco smoking as fixed covariates, and NLR, hypertension, SBP, total cholesterol and HDL as time dependent covariates. Model D included Model C plus intravenous drug use as fixed covariate and CD4 cell count and antiretroviral therapy as time dependent covariates. Model E included Model D plus GFR. Abbreviations: HR, hazard ratio; 95% CI, 95% confidence interval; NLR, neutrophil to lymphocyte ratio; SBP, systolic blood pressure, HDL, high density lipoprotein; GFR, Glomerular filtration rate estimated using the Modification of diet in renal disease (MDRD) formula.
(DOCX)

## Abbreviations

AIC criterion: Akaike’s information criterion
AUC: area under the curve
cART: combined antiretroviral therapy
CHD: coronary heart disease
CIs: confidence intervals
CRP: C-reactive protein
CUORE score: Cardiovascular risk score for the Italian general population
CVD: cardiovascular disease
FRS: Framingham Risk Score
GFR: glomerular filtration rate (formula
HBV: Hepatitis B Virus
HCV: Hepatitis C Virus
HDL: High density lipoprotein
HR: hazard ratios
LHA: Local Health Authority
MDRD formula: modification of diet in renal disease formula
NLR: Neutrophil to lymphocyte ratio
ROC curve: receiver operating characteristics curve
SBP: systolic blood pressure
SD: Standard deviation
SIR: standard incidence ratio
