# Supplementary figures and images for: Neutrophil to Lymphocyte Ratio and Cardiovascular Disease Incidence in HIV-Infected Patients: A Population-Based Cohort Study
Source: PLoS One. 2016 May 5;11(5):e0154900. doi: 10.1371/journal.pone.0154900 (PMC4858273; doi:10.1371/journal.pone.0154900)

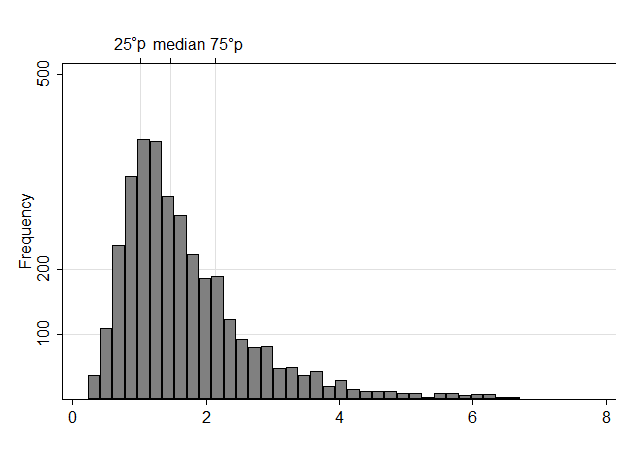

Supplement: S1 Fig — Abbreviation: NLR, neutrophil to lymphocyte ratio. (TIF) [file pone.0154900.s001.tif]
