# Supplementary material for: Neutrophil to Lymphocyte Ratio and Cardiovascular Disease Incidence in HIV-Infected Patients: A Population-Based Cohort Study
Source: PLoS One. 2016 May 5;11(5):e0154900. doi: 10.1371/journal.pone.0154900 (PMC4858273; doi:10.1371/journal.pone.0154900)
Supplement: S1 Table — Model A included NLR. Model B included NLR, age, sex, hypertension, diabetes, tobacco smoking, SBP, total cholesterol and HDL. Model C included age, sex, diabetes and tobacco smoking as fixed covariates, and NLR, hypertension, SBP, total cholesterol and HDL as time dependent covariates. Model D included Model C plus intravenous drug use as fixed covariate and CD4 cell count and antiretroviral therapy as time dependent covariates. Model E included Model D plus GFR. Abbreviations: HR, hazard ratio; 95% CI, 95% confidence interval; NLR, neutrophil to lymphocyte ratio; SBP, systolic blood pressure, HDL, high density lipoprotein; GFR, Glomerular filtration rate estimated using the Modification of diet in renal disease (MDRD) formula. (DOCX) [file pone.0154900.s002.docx]

**S1 Table**. Hazard ratio of CVD event for NLR as a continuous variable using various Cox proportion hazard models.

| **Model** | **n** | **HR** | **95% CI** | **p value** |
| --- | --- | --- | --- | --- |
| **A** | **3454** | 1.19 | 1.07-1.32 | 0.001 |
| **B** | **1980** | 1.21 | 1.03-1.43 | 0.023 |
| **C** | **2499** | 1.26 | 1.10-1.43 | 0.001 |
| **D** | **2436** | 1.27 | 1.10-1.46 | 0.001 |
| **E** | **1742** | 1.26 | 1.08-1.48 | 0.004 |

Model A included NLR.

Model B included NLR, age, sex, hypertension, diabetes, tobacco smoking, SBP, total cholesterol and HDL.

Model C included age, sex, diabetes and tobacco smoking as fixed covariates, and NLR, hypertension, SBP, total cholesterol and HDL as time dependent covariates.

Model D included Model C plus intravenous drug use as fixed covariate and CD4 cell count and antiretroviral therapy as time dependent covariates.

Model E included Model D plus GFR.

Abbreviations: HR, hazard ratio; 95% CI, 95% confidence interval; NLR, neutrophil to lymphocyte ratio; SBP, systolic blood pressure, HDL, high density lipoprotein; GFR, Glomerular filtration rate estimated using the Modification of diet in renal disease (MDRD) formula.
